# Correction: How Many Political Parties Should Brazil Have? A Data-Driven Method to Assess and Reduce Fragmentation in Multi-Party Political Systems

**DOI:** 10.1371/journal.pone.0147656

**Published:** 2016-01-21

**Authors:** Pedro O. S. Vaz de Melo

## Notice of Republication

This article was republished on January 4, 2016, to correct post-publication errors that the author discovered in the underlying data and analysis. The errors were caused by a bug in the software analysis tool, which replicated data and therefore skewed the presented results in Table 1. The bug has been fixed. These errors did not affect the overall conclusions of the paper. After re-analysis, the corrected article was editorially reviewed and accepted, and due to the extent of the errors, the article was republished. Please download this article again to view the correct version. The originally published, uncorrected article and the republished, corrected article are provided here for reference.

## Supporting Information

S1 File Originally published, uncorrected article. (PDF)

S2 File Republished, corrected article. (PDF)
